# Photomorphogenesis of *Myxococcus macrosporus*: new insights for light-regulation of cell development

**DOI:** 10.1007/s43630-024-00635-1

**Published:** 2024-09-19

**Authors:** Kinga B. Graniczkowska, Dorina Bizhga, Moraima Noda, Viridiana Leon, Niharika Saraf, Denisse Feliz, Gaurav Sharma, Angela C. Nugent, Mitchell Singer, Emina A. Stojković

**Affiliations:** 1 Department of Microbiology and Molecular Genetics, College of Biological Sciences, University of California-Davis, One Shields Ave, Davis, CA 95616, USA; 2 Department of Biology, Northeastern Illinois University, 5500 N. St. Louis Ave., Chicago, IL 60625, USA; 3 Department of Biotechnology, Indian Institute of Technology Hyderabad, Sangareddy, Telangana 502284, India

## Abstract

Myxobacteria are non-photosynthetic bacteria distinguished among prokaryotes by a multicellular stage in their life cycle known as fruiting bodies that are formed in response to nutrient deprivation and stimulated by light. Here, we report an entrained, rhythmic pattern of *Myxococcus macrosporus* fruiting bodies, forming consistently spaced concentric rings when grown in the dark. Light exposure disrupts this rhythmic phenotype, resulting in a sporadic arrangement and reduced fruiting-body count. *M. macrosporus* genome encodes a red-light photoreceptor, a bacteriophytochrome (BphP), previously shown to affect the fruiting-body formation in the related myxobacterium *Stigmatella aurantiaca*. Similarly, the formation of *M. macrosporus* fruiting bodies is also impacted by the exposure to BphP—specific wavelengths of light. RNA-Seq analysis of *M. macrosporus* revealed constitutive expression of the *bphP* gene. Phytochromes, as light-regulated enzymes, control many aspects of plant development including photomorphogenesis. They are intrinsically correlated to circadian clock proteins, impacting the overall light-mediated entrainment of the circadian clock. However, this functional relationship remains unexplored in non-photosynthetic prokaryotes. Genomic analysis unveiled the presence of multiple homologs of cyanobacterial core oscillatory gene, *kaiC*, in various myxobacteria, including *M. macrosporus*, *S. aurantiaca and M. xanthus*. RNA-Seq analysis verified the expression of all *kaiC* homologs in *M. macrosporus* and the closely related *M. xanthus*, which lacks *bphP* genes. Overall, this study unravels the rhythmic growth pattern during *M. macrosporus* development, governed by environmental factors such as light and nutrients. In addition, myxobacteria may have a time-measuring mechanism resembling the cyanobacterial circadian clock that links the photoreceptor (BphP) function to the observed rhythmic behavior.

## Graphical Abstract

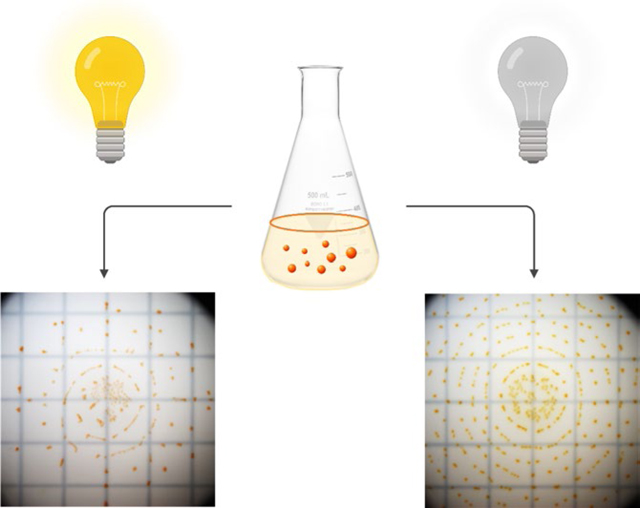

## 1 Introduction

Myxobacteria are non-photosynthetic, soil bacteria distinguished for the starvation-induced multicellular stage of their life cycle. Individual cells aggregate to form fruiting bodies consisting of stalks bearing sporangioles. Within the sporangioles reside heat- and desiccation-resistant myxobacterial spores (myxospores) [1]. The morphology of fruiting bodies is highly variable between species and the genetic mechanisms involved in the formation of these structures are not well understood, with the exception of *Myxococcus xanthus,* the model organism for *Myxococcales* [2].

While fruiting-body development for all known myxobacteria can be initiated by nutrient deprivation, other environmental factors such as light are also known to affect the developmental program. In *M. xanthus*, exposure to light activates carotenoid synthesis [3] white light inhibits the mound-forming stage of the fruiting-body development [4]. The molecular mechanism of inhibitory effect of light on development of *M. xanthus* is unknown but the mechanism of carotenoid biosynthesis and the *carQRS* regulatory system are well-characterized (for review see [5]). Interestingly, in some species, such as *Stigmatella aurantiaca*, exposure to light is required for fruiting-body development [6, 7]. In this manuscript, we examine the photomorphogenesis of *Myxococcus macrosporus* HW-1 (previously known as *Myxococcus fulvus* HW-1 and *Corallococcus macrosporus* HW-1), the closest relative to well-characterized *M. xanthus,* involving the effects of light on the fruiting-body formation.

The photomorphogenic response of fruiting-body formation in *S. aurantiaca* was reported in the late 1970s, decades prior to the first characterization of protein photoreceptors from eubacteria [7]. Interestingly, *S. aurantiaca* contains two bacterial red-light photoreceptors (bacteriophytochromes denoted as BphPs) [8] and a single blue light photoreceptor (photoactive yellow protein, PYP). BphPs absorb light at 700 nm (red) and 750 nm (far-red) and undergo reversible photoconversion between the two distinct states, denoted as *P*_r_ and *P*_fr_, for red and far-red light absorbing, respectively. The reversible photoconversion is essential in regulating activity of the C-terminal enzymatic domain of BphPs, usually a histidine kinase (HK). Both BphPs from *S. aurantiaca* contain C-terminal HKs that are covalently linked to N-terminal photosensory core modules (PCMs), essential for sensing the red and far-red light. The function of BphPs in photosynthetic bacteria is to modulate the synthesis of light-harvesting complexes [9–11]. However, the physiologic role of BphPs in the non-photosynthetic bacteria remains largely unknown, although in some species they are involved in swarming motility, gene transfer and carotenoid synthesis [12, 13]. Recently, Malla et al., demonstrated that BphP from *S. aurantiaca* is a HK regulated by red and far-red light[14]. Photomorphogenesis of *S. aurantiaca* was observed when incubated on starvation media under wavelengths of light specific to BphP function, 700 nm (red) and 750 nm (far-red) [6]. Although readily developing at 750 nm, the fruiting bodies of *S. aurantiaca* failed to form when exposed to 700 nm light. This photomorphogenic response in the formation of fruiting bodies of *S. aurantiaca* led us to hypothesize that a BphP is also involved in the development of *M. macrosporus* that contains one BphP-encoding gene with C-terminal HK domain and entirely lacks genes coding for PYP.

*M. macrosporus* as the closest genetic relative to *M. xanthus* (*M. xanthus* lacks BphP and PYP-coding genes) is an ideal model to study the function of BphP photoreceptors in the fruiting-body formation. Interestingly, the fruiting bodies of *M. macrosporus* formed concentric rings in in the dark, over 14 days, resembling oscillatory growth patterns of the fungus *Neurospora crassa* [15, 16]. Exposure to light, including red and far-red light, disrupted this rhythmic phenotype suggesting a possible BphP function. The fungus *Neurospora crassa* displays similar oscillatory growth pattern under starvation conditions that is disrupted by light exposure. Published in 2009, the rhythmic phenotype of *N. crassa* leads to further circadian rhythm studies in other microorganisms [17–20]. The circadian clock is a molecular mechanism that evolved with the organisms’ adaptations to the Earth’s daily oscillation of light and external temperature [21]. The first bacterial circadian clock was demonstrated in cyanobacteria. The oscillator system in cyanobacteria comprises of three proteins KaiA, KaiB, and KaiC. Daily changes in KaiC phosphorylation and alternating interactions with KaiA at daytime, or KaiB during the night, manage the whole metabolism of cyanobacteria [22]. Homologs of KaiB and KaiC proteins can be found in many other bacteria, such as purple bacterium *Rhodopseudomonas palustris* [23]; however, KaiA proteins have only been identified in cyanobacteria [24]. KaiC homologues are found in multiple copies in many Cyanobacteria, Bacteroidetes, Proteobacteria, and even Archaea [24–26], but the function of this protein is still unclear [21, 22]. Experiments investigating the role of KaiC in *Legionella pneumophila* demonstrated its relevance in stress response and adaption to changes in the environment [27]. A recent study of environmental *Pseudomonas* species showed that KaiC proteins are involved in osmotic and oxidative stress resistance and in biofilm production, without evidence for circadian function [28].

Conversely, a circadian rhythmicity of gene expression has been described for two non-photosynthetic bacteria *Klebsiella aerogenes* [18–20], and *Bacillus subtilis* [29, 30], even though these bacteria do not encode any of the Kai proteins. In cyanobacteria, a phytochrome-like protein CikA is directly involved in regulating the circadian clock genes [31]. As some Myxobacteria encode BphPs in tandem with KaiC genes, we hypothesize that both of these genes might be involved in regulating fruiting-body formation. Moreover, *M. macrosporus HW-1* has been isolated from the coastal microbial mat, and microbial mats demonstrate circadian rhythmicity in their gene expression [32, 33]. Coastal microbial mats exhibit 24-h cycles, wherein processes such as photosynthesis, respiration, fermentation, nitrogen fixation, and microbial migration occur at specific times. These processes are influenced by the alternating periods of light and darkness as well as the tidal fluctuations [32, 34].

This work demonstrates previously uncharacterized light- and nutrient-dependent development of fruiting-body formation in *M. macrosporus* HW-1. The periodic appearance of concentric rings involving *M. macrosporus* fruiting bodies has not been reported for any other myxobacteria. We use RNA sequencing, RNA-Seq, to determine the expression of genes encoding a BphP, circadian clock proteins and carotenoid biosynthesis in *M. macrosporus*, dependent on the presence of light as an environmental signal. In addition, we performed comparative evolutionary analysis of cyanobacterial and myxobacterial KaiC protein sequences. Our results describe the photomorphogenesis of *M. macrosporus*, the function of BphP in non-photosynthetic myxobacteria, and the potential role that circadian clock proteins play in this complex development cycle.

## 2 Methods

### 2.1 Bacterial strains, growth, and sampling conditions

*M. xanthus* DK1622 and *M. macrosporus* HW-1 strains were grown in CTTYE broth (1% casitone, 10 mM Tris–HCl pH 7.6, 1 mM KH_2_PO_4_, 8 mM MgSO_4_) for 36 h at 33 °C in constant darkness or in 12:12 light–dark (LD) cycle, with white LED light (380 lx). Vegetative cells were cultivated and collected in four replicates for illuminated and dark samples although we excluded one *M. macrosporus* replicate cultivated in the dark due to insufficient ribosomal RNA depletion. Transcription was stopped immediately upon cell collection using stop solution (5% acidic phenol in ethanol [35]). Bacterial cell pellets obtained after centrifugation at 3000 rpm at 4 °C were frozen in liquid nitrogen, and stored at − 80 °C until the mRNA extraction.

### 2.2 Fruiting-body formation and image processing

Liquid CTTYE media was inoculated with *M. macrosporus* and incubated for 2 days at 33 °C, 150 rpm. Following centrifugation, vegetative cells were resuspended and washed three times with 0.1 M phosphate buffer pH 8.0 and applied in 10 μL aliquots to three types of starvation media: (a) 1.5% Wasseragar [6] prepared with Bactoagar [(gel strength 680 g/cm^2^), Apex, Cat #: 20–275]; (b) 1.5% Wasseragar prepared with Ultrapure Noble Agar [(gel strength 700 g/cm^2)^, Thermo Scientific^™^, J10907.36]; and c) 5% Wasseragar prepared with Bactoagar. Pictures were captured using 10× magnification by a Nikon SMZ800 dissecting microscope. Binary images of *M. macrosporus* fruiting bodies on filter paper or agar were created with Image J [36]. To create the images, they were loaded into ImageJ, and the tritanopia selection was applied by navigating to “Image,” “Color,” “Dichromacy,” and selecting “Tritanope.” Color deconvolution was performed from the selected gridlines of the filter paper. The settings were modified until the black and white particles matched the size of the original images. Finally, the images were cleaned up using a 50–100 pixel black or white paintbrush to remove noise from the edges and any unwanted center spots.

### 2.3 Phylogenetic analysis

KaiC-like proteins encoded in all available genomes from Myxococcales order were identified via homology searches using the *S. elongatus* PCC7942 KaiC protein (AAM82686) as a query and applying an e-value cut-off of 1E^−20^. Subsequently, characteristic domains (PF06745; ATPase) were identified in the candidate proteins. Phytochrome proteins (BphPs) were identified via homology searches using *Pseudomonas* bacteriophytochrome protein (OPF44805) against all order Myxococcales organisms keeping an e-value cut-off of 1E^−20^ followed by their domain (PF00360; PHY) identification. Twenty-five conserved housekeeping genes were identified in all Myxococcales organisms, followed by alignment of each gene homolog using MUSCLE (v5.1.linux64) [37]. All alignment blocks were concatenated to form a larger alignment for all organisms. This alignment was used to generate a maximum likelihood tree using RaxML using the best model selected as predicted by IQ-TREE (v2.1.4-beta) [38]. iTOL was used for visualization and mapping of Phytochrome and KaiC protein counts [39]. Myxobacterial KaiC query sequences were subjected to homology search against the NR protein sequence database using command-line DIAMOND v2.1.0.154 [40] blastp to find the top 100 matches for each query sequence. All resultant hits were used to identify unique hits (1109 sequences) which were further used for the phylogenetic analysis. Multiple-sequence alignment was carried out using MUSCLE (v5.1.linux64) [37], model selection was done using IQ-TREE (v2.1.4-beta) [38] and then FastTree (v2.1.11) [41] was used to build a maximum likelihood phylogeny tree using the LG model and 100 bootstraps. The visualization and phylum-level taxonomy mapping were performed on iTOL [39].

### 2.4 RNA isolation and ribosomal RNA depletion

Total RNA was isolated using the hot phenol method followed by ribosomal RNA (rRNA) depletion implementing previously validated protocol [42]. Briefly, approximately 4 μg total RNA was mixed with 10 × hybridization buffer (1 M Tris–HCl pH 7.0, 2 M NaCl, RNase-free H2O), 2 μL 16S and 23S synthetic DNA oligonucleotide probes (developed by our lab previously [42]), and RNase-free H_2_O. Hybridization was performed at 45 °C for 5 min. The hybridization reaction was treated with thermostable RNaseH (NEB, M0523S) and corresponding buffer, followed by incubation at 45 °C for 40 min. The reaction was transferred to ice and then treated with RNase-free Dnase I (NEB, M0303S) according to manufacturer’s protocol. The Dnase I treated samples were shifted to ice then followed with an Agencourt RNAClean^™^ XP bead cleanup (Beckman Coulter, Brea, CA) with a 1.8× volume of beads to sample as per manufacturers protocol. RNA samples depleted of rRNAs were eluted off the beads in RNase-free H_2_O.

### 2.5 Library preparation and sequencing

Following depletion of total rRNA, the remaining RNAs were used as input to prepare sequencing libraries. Libraries were generated using NEBNext^®^ Ultra^™^ II Directional RNA Library Prep Kit (NEB #E7765), according to the manufacturer’s protocol without PolyA selection and rRNA depletion steps. Sixteen strand-specific RNA libraries were sequenced using the Illumina HiSeq platform.

### 2.6 Bioinformatics

FastQC (version v0.11.8) was applied to check the quality of raw reads. Trimmomatic (version v0.38) was applied to cut adaptors and trim low-quality bases with default setting. STAR Aligner version 2.7.1a was used to align the reads. Picard tools (version 2.20.4) were applied to mark duplicates of mapping. The StringTie version 2.0.4 was used to assemble the RNA-Seq alignments into potential transcripts and calculate Transcripts Per Million (TPM) values [43]. It is a normalized estimation of gene expression based on the RNA-Seq data, which considers the gene length as well as relative expression in normalization. The feature Counts (version 1.6.0)/HTSeq was used to count mapped reads for genomic features. The De-Seq2 (version 1.14.1) was used to do the differential analysis. Data can be downloaded from the NCBI database under accession number GSE244575.

## 3 Results

### 3.1 Differences in spatial organization of *M. macrosporus* fruiting-bodies

**Fig. 1:**
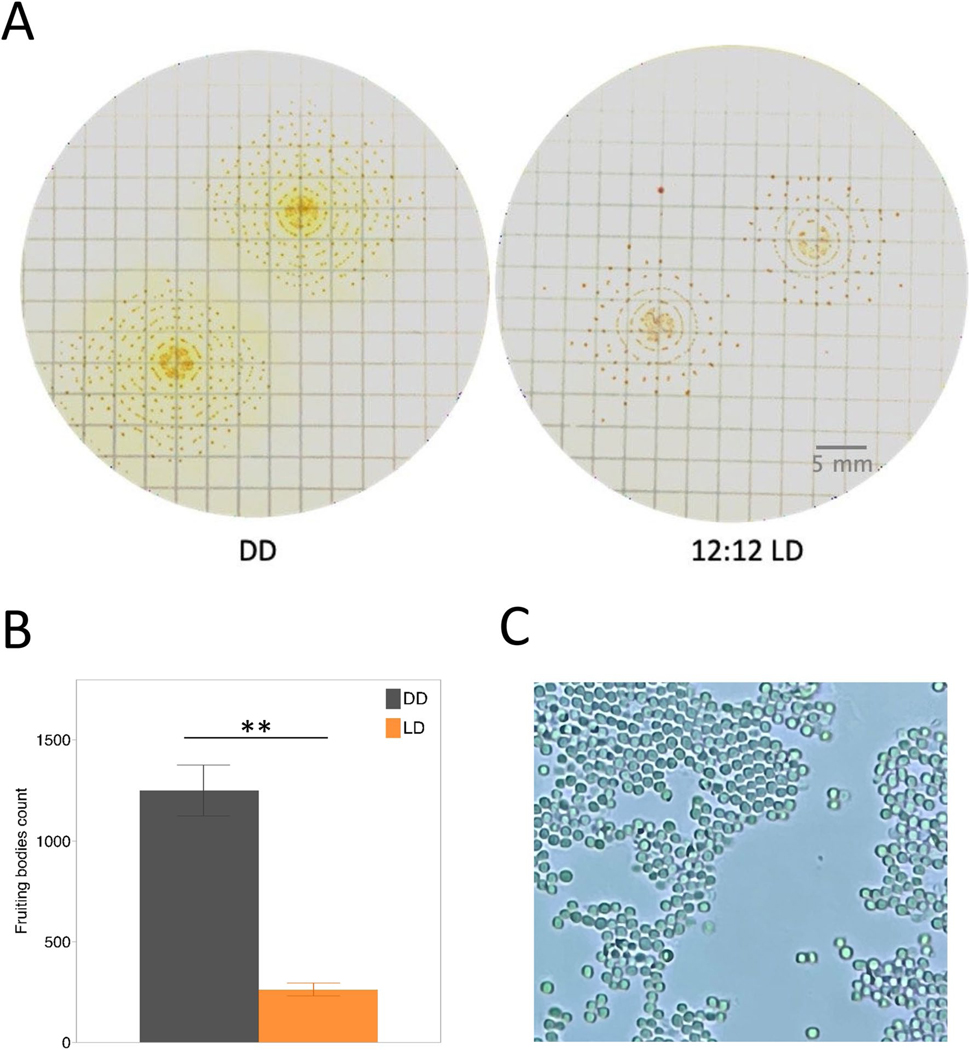
Light-sensitive fruiting-body formation in *M. macrosporus* **A** on filter paper in continuous darkness (DD) on the left, and oscillating light (12:12 LD) on the right. Images were taken 14 days post-inoculation. **B** Quantitative analysis of the fruiting-body counts formed within the concentric rings of *M. macrosporus* in the constant darkness and LD cycle. Statistical differences were determined by the two-tailed Student’s *t* test. **, *t* < 0.0061. **C** *M. macrosporus* spores isolated from the fruiting bodies grown on starvation media for 14 days in the constant darkness. Image was captured using light microscope and ×100 oil immersion lens

**Fig. 2:**
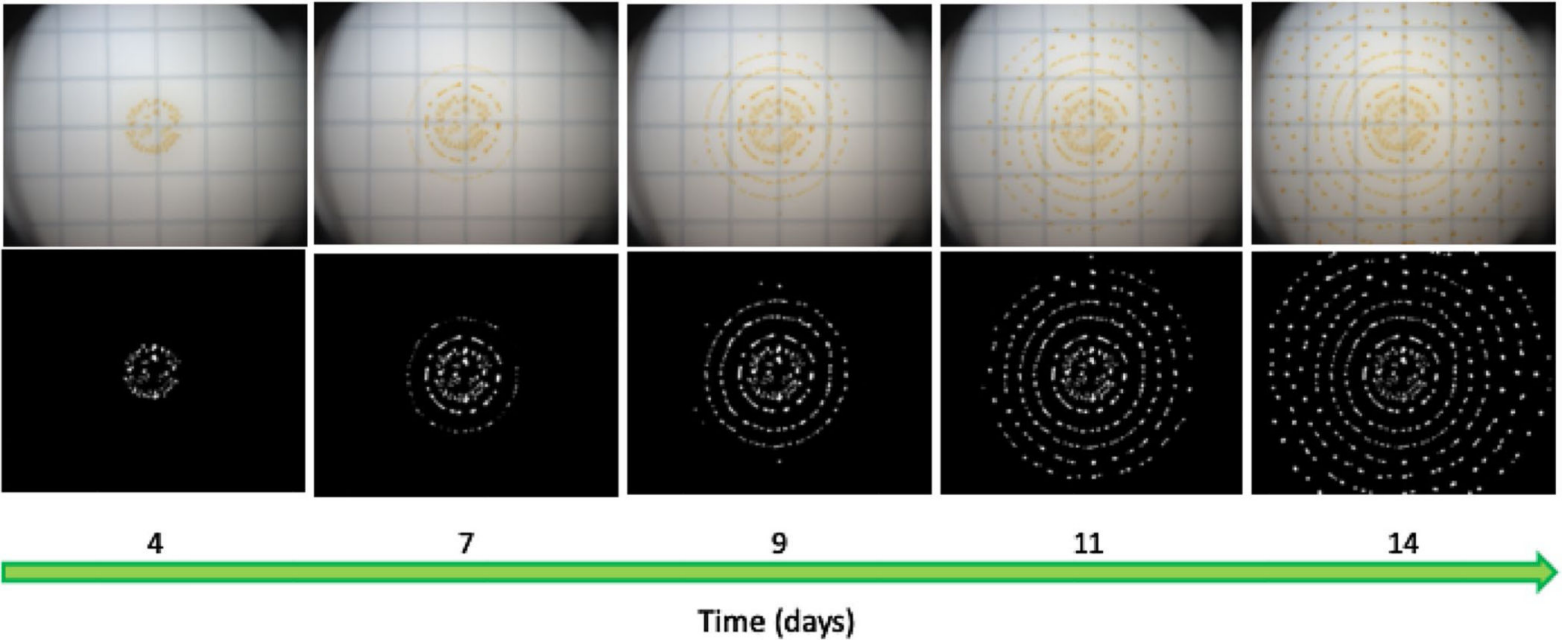
*M. macrosporus* fruiting-body formation on filter paper between day 4 and day 14 post-inoculation (PI) on starvation media viewed top to bottom in white light (top) and the corresponding binary images (bottom). Cells were incubated in constant darkness starting from day 0 with images of fruiting bodies captured on the selected days of development

The fruiting bodies of *M. macrosporus* form structured concentric rings, when grown in constant darkness (Figs. 1A and 2), a growth pattern that has not been described before for any myxobacteria. *M. macrosporus* fruiting bodies start forming 2 days post-inoculation of vegetative cells on starvation agar media. By day 4, fruiting bodies appear as concentric rings around the original aliquot of the liquid culture (Fig. 2) when plates are incubated at 30 °C. These rings are periodic in nature, forming with consistent spacing during incubation in the dark (DD) (Fig. 2). The concentric ring pattern of *M. macrosporus* fruiting bodies is disrupted by light, specifically 12:12 LD cycle or constant light (Fig. 1A and Fig. S1). Furthermore, the LD cycle reduces the number of fruiting bodies (Fig. 1B and Fig. S1) in comparison to fruiting bodies formed in total darkness (DD).

In the laboratory setting, the ring formation of *M. macrosporus* fruiting bodies occurs in an oscillatory manner consistent with light exposure. Over the span of 14 days, multiple concentric rings form as long as the rings from the neighboring cluster of fruiting bodies are not within the critical distance (Fig. 1A). This avoidance behavior is observed in both light and dark conditions (Fig. 1A). Furthermore, fruiting-body formation of *M. macrosporus* is impacted by exposure to BphP-specific wavelengths of lights, red and far-red, with fewer rings and fewer fruiting bodies being present in the red light (Fig. S2). On starvation agar media, on day 11 to day 14, fruiting bodies grown in constant light, and/or 12:12 LD cycle are more pigmented than the ones grown in constant darkness (Fig. 1A and Fig S1). It should be noted that the fruiting bodies of *M. xanthus* and *S. aurantiaca* do not form multiple concentric rings. Myxospores are obtained from the fruiting bodies of *M. macrosporus* within the outer rings and in the center within the original liquid culture application regardless of the light exposure (Fig. 1C). Spores start germinating within 24 h after placement on rich CYE media (data not shown).

**Fig. 3:**
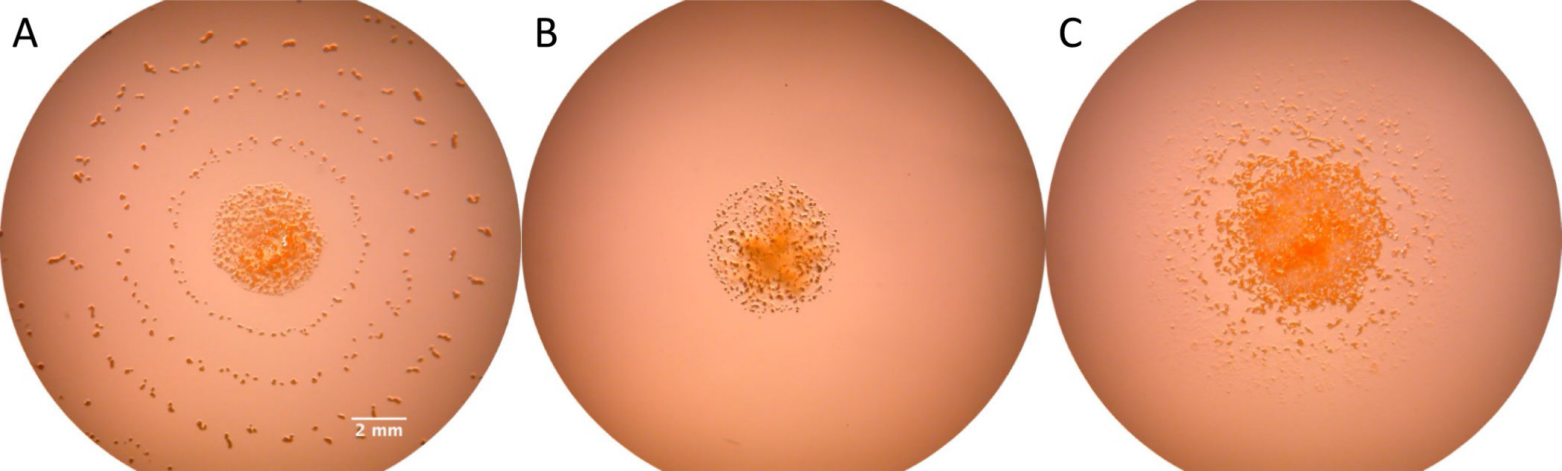
*M. macrosporus*-fruiting bodies formed on starvation agar media 14 days post-inoculation. **A** The agar plate was prepared with standard bactoagar (1.5%), **B** purified agar (1.5%), and **C** purified agar (1.5%) with addition of 0.1% of casamino acids (magnification ×10)

To investigate whether the trace amounts of nutrients present in the standard bactoagar affect the observed phenotype of fruiting bodies of *M. macrosporus*, we compared two different kinds of agar with almost identical gel strength, bactoagar (with trace amount of nutrients) and purified agar (with no specific measurable nutrients). *M. macrosporus* fruiting bodies did not form rings on 1.5% purified agar, which suggests that the concentric ring formation requires a trace amount of nutrients present in bactoagar (Fig. 3A and B). Therefore, we prepared media with purified agar and 0.1% of casamino acids to test if this mixture will recover the concentric ring phenotype observed with bactoagar. Indeed, additional fruiting bodies formed around the initial inoculation spot; although multiple concentric rings were absent (Fig. 3C). To test whether the observed concentric ring formation depends on gel strength of solid media, starvation petri plates were prepared with different concentrations of bactoagar (Fig. S3). We observed that the bactoagar concentration and the distance between the rings of *M. macrosporus* fruiting bodies were inversely related: the lower the bactoagar concentration, the greater the distance between the rings (Fig. S3). At 5% bactoagar concentration, the fruiting bodies are irregularly distributed (Fig. S3B) throughout the petri dish without any distinguishable pattern formation.

### 3.2 Expression of bacteriophytochrome and carotenoid genes

**Fig. 4:**
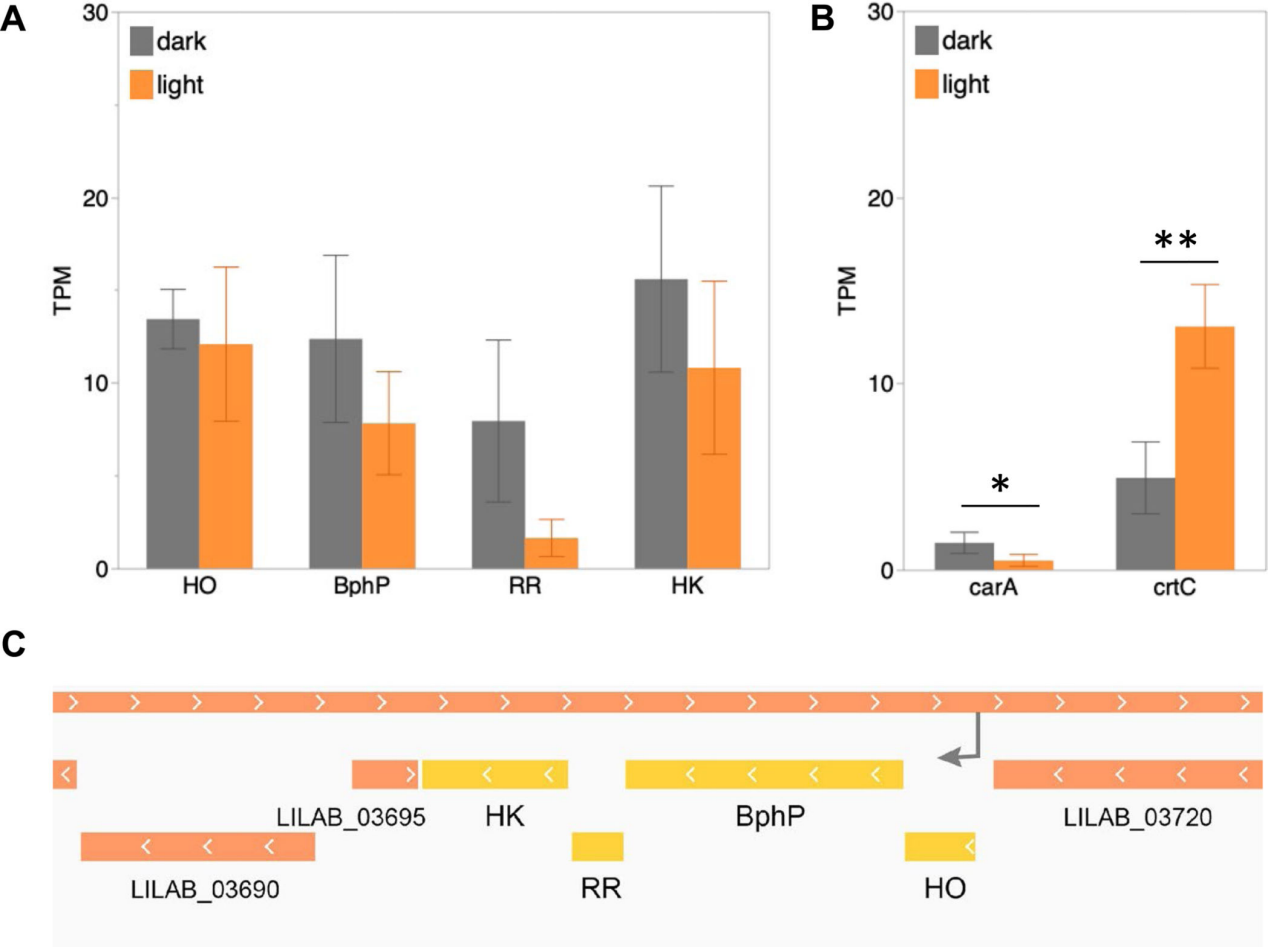
Expression of operons involving bacteriophytochrome and carotenoid synthesis in vegetative cells of *M. macrosporus* collected after 36 h of incubation. Cells were cultivated in the darkness or in the presence of white light. **A** Bar-graph illustrating TPM values for Bacteriophytochrome (BphP), heme oxygenase (HO), response regulator (RR) and a histidine kinase (HK). Each error bar refers to 1 standard deviation from the mean. **B** Bar-graph illustrating TPM values for *carA* and *crtC* genes from *M. macrosporus;* *, *p* < 0.05; **, *p* < 0.001. **C** Genomic organization of *M. macrosporus* HW-1 of BphP operon, depicting HO, BphP, RR, and HK in yellow while the gray arrow indicates the promoter upstream of the operon

Based on the light-induced differences in *M. macrosporus* fruiting-body arrangement, we hypothesized that the single BphP-gene is involved in the generation of these structures. To characterize the gene expression of the entire BphP operon, RNA-Seq was performed on vegetative cells of *M. macrosporus*, cultivated in liquid culture under 12:12 LD or in the darkness. *M. macrosporus* BphP operon, consists of four genes: bacteriophytochrome (BphP; LILAB_03710/ LILAB_RS03635), heme oxygenase (HO; LILAB_03715/ LILAB_RS03640), response regulator (RR; LILAB_03705/ LILAB_RS03630) and an additional Histidine Kinase (LILAB_03700/ LILAB_RS03625). HO is required for the synthesis of biliverdin, an organic open-chain tetrapyrrole that is required for BphP to respond to light. The RR is a downstream component of the BphP regulated two-component signaling pathway. Most BphPs, including *M. macrosporus* and *S. aurantiaca* BphPs contain a HK as the C-terminal enzymatic domain that will phosphorylate a downstream RR protein to ultimately trigger a gene expression. Statistical analysis indicated that light did not affect expression of any of the genes within the BphP operon (Fig. 4A). When normalized counts were compared for individual genes within the BphP operon, the HK was the highest followed by HO and BphP, with the lowest values detected for the RR.

Due to differences in the pigmentation of fruiting bodies exposed to light in comparison to those in the dark, we analyzed expression of the carotenoid biosynthesis genes and the regulatory *carQRS* operon. Although the expression of above-mentioned genes in *M. xanthus* was not affected by the light, for *M. macrosporus* we observed differential expression of *crtC*, and *carA genes* between light and dark conditions (Fig. 4B). *CarA* that encodes a transcriptional factor, was upregulated in cultures cultivated in the dark while *crtC* that encodes a hydroxyneurosporene synthase which hydroxylates γ-carotene [5], was upregulated in light-illuminated cultures.

The gene expression of *M. macrosporus* and *M. xanthus* analyzed by the RNA-Seq experiments involved vegetative cells grown in liquid culture in contrast to fruiting bodies, induced by starvation. It is possible that light-induced changes in pigmentation of *M. macrosporus* fruiting bodies (Fig. 1A and S1A) are most likely due to activation of the homologs of *M. xanthus carQRS* system [44].

### 3.3 Circadian clock genes in myxobacteria

**Fig. 5:**
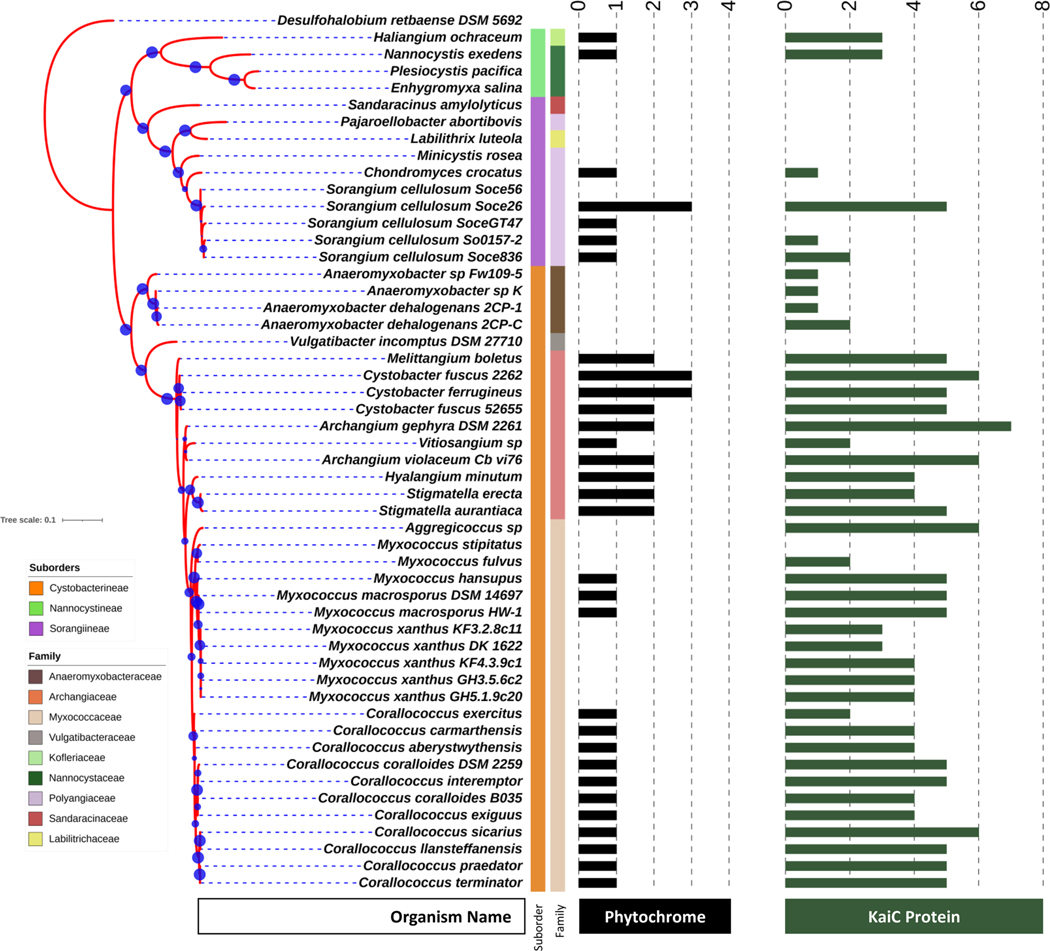
Genomic distribution of putative phytochrome and KaiC protein homologs within order Myxococcales as highlighted and mapped on the phylogenetic tree. Blue circles represent the bootstrap values used in the generation of phylogenetic tree sampling. The central strips represent the suborder and family level taxonomy of each organism as also mentioned in the left-bottom corner

**Fig. 6:**
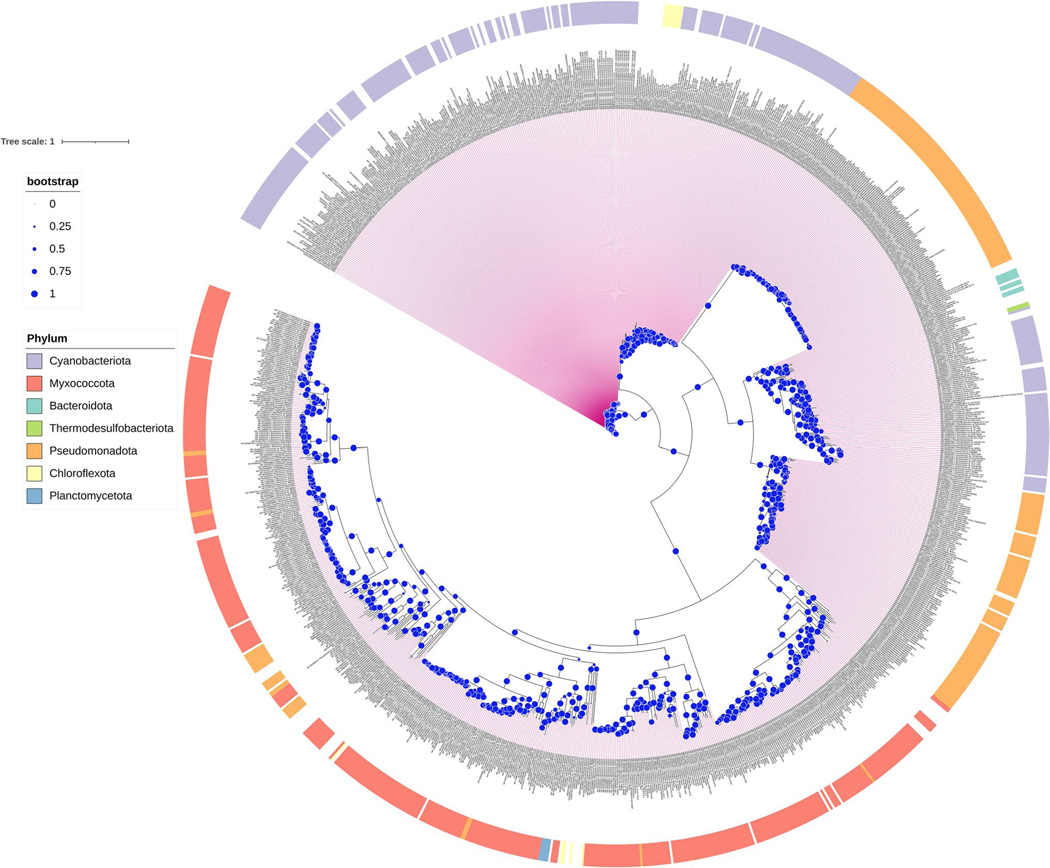
Evolutionary relationships of the myxobacterial KaiC proteins. Maximum likelihood phylogenetic tree depicting myxobacterial KaiC homologs and their closest evolutionary KaiC homologs from different bacterial species. Blue color circles display branch support values according to the left-top legends for bootstrap values. The outer circle represents the taxonomy of each homolog as mapped using the iTOL

Given the oscillatory formation of the concentric rings of *M. macrosporus* fruiting bodies, we conducted comparative genomic studies focused on potential myxobacterial homologs of the well-known cyanobacterial KaiABC complex [22]. The majority of the myxobacteria encode at least one putative gene encoding for *kaiC* homolog; however, none of them encode *kaiA* and only a few have *kaiB* gene (Fig. 5), in accordance with previously published reports [24, 25]. The function of myxobacterial *kaiC* genes have not been studied to date, although multiple copies of *kaiC* genes exist, ranging from 1–7 per genome of various myxobacterial strains. *M. macrosporus* (NC_015711) and *S. aurantiaca* encode 5 *kaiC* protein homologs each (Figs. 5 and 6). Interestingly *M. xanthus* that lacks the *bphP* gene, encodes 3 *kaiC* homologs. We examined the diversity of *kaiC* genes across all sequenced genomes of various myxobacteria (Fig. 5) and compared their phylogenetic evolution. We identified conserved phosphorylation sites based on cyanobacterial KaiC proteins to better understand their functional diversity (Fig. S4). Our analysis revealed that myxobacterial KaiC homologs constitute a distinct clade, away from another large clade having KaiC protein from phylum Pseudomonadota and Cyanobacteriota (blue-green algae) including the well-known model organism, *Synechococcus elongatus*. The presence of multiple homologs among the myxobacterial genomes can be correlated to gene duplication events within each organism for functional diversification [45]. Myxobacteria harbor large genomes ranging from 9–16 Mbs; the model organism, i.e., *M. xanthus* (9.14 Mb) contains ~ 8000 genes among which more than 1500, have been expanded using duplication events followed by functional diversification [45, 46]. Multiple-sequence alignment of the identified myxobacterial KaiC proteins with cyanobacterial KaiC proteins revealed the presence of important phosphorylation sites. *S. elongatus* KaiC has two conserved phosphorylation sites, i.e., involving Ser431 and Thr432. Most myxobacterial KaiCs (~ 60 proteins) contain only a single-phosphorylation site, either at serine (Ser) or threonine (Thr); however, nine myxobacterial KaiC proteins have both Ser and Thr sites (Fig. S4). Recent studies conducted by the Kondo research group have demonstrated that a single-phosphorylation site at Ser431 is sufficient for generating a temperature-compensated circadian period [47]. Fifty myxobacterial KaiCs contain no phosphorylation sites, in contrast to the canonical cyanobacterial KaiC (Fig. S4) [48]. This lack of phosphorylation sites leads to the question as to how these distantly related KaiC homologues act. Based on the phylogenetic analysis, myxobacterial KaiCs are distantly related to cyanobacterial circadian oscillatory genes. In addition, these homologs are composed of different numbers of amino acids, with cyanobacterial KaiC_Se_ containing 519 aa, and homologs from *M. xantus* and *M. macrosporus* being shorter, ranging between 470 and 514 aa. Furthermore, myxobacterial KaiC proteins have amino acid sequence homology to the 10-residue sequence A-loop from *S. elongatus*, which is the binding site for KaiA [49]. The B-loop sequence for the binding of KaiB protein is missing (Fig. S5).

**Fig. 7:**
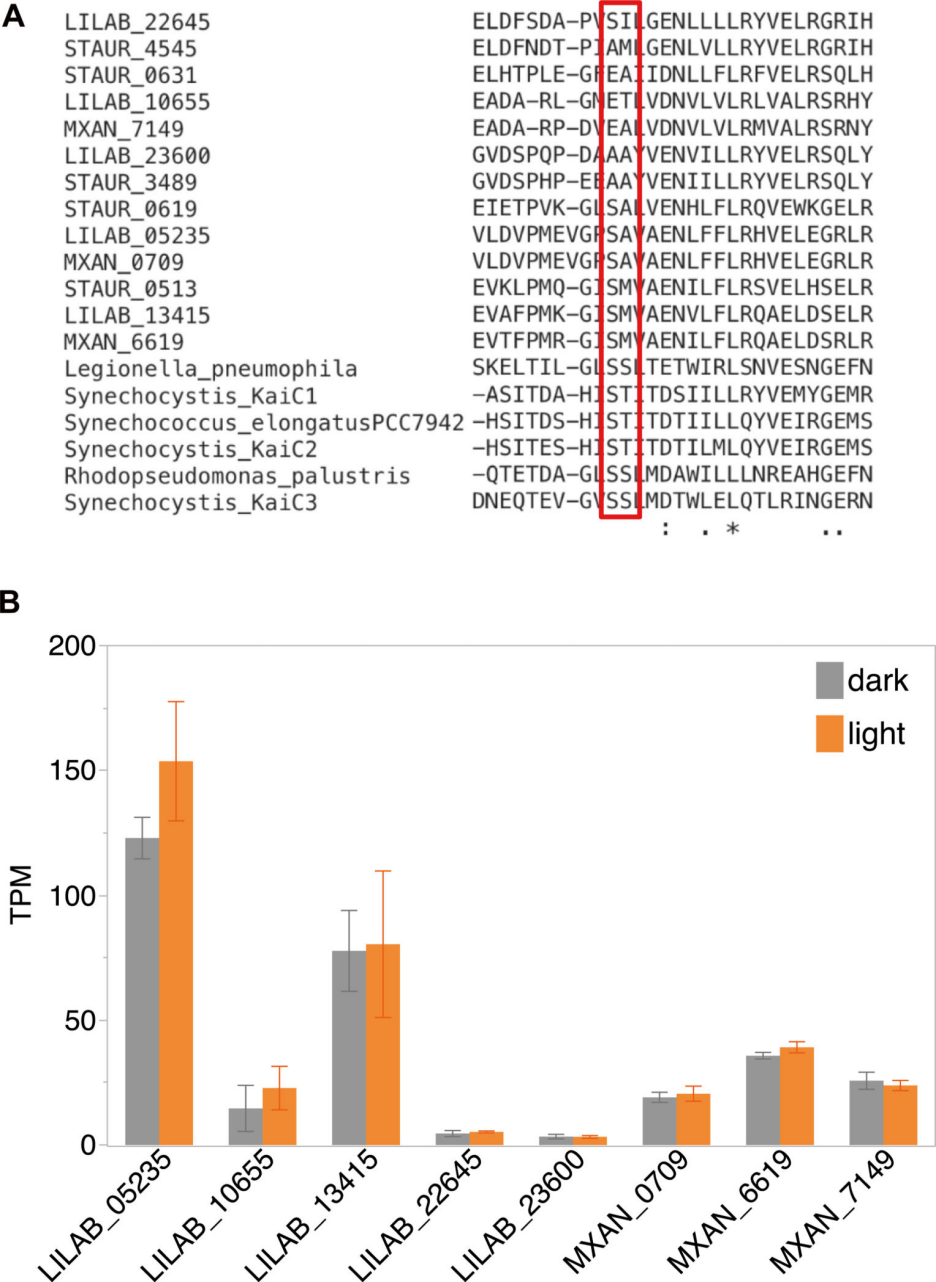
Predicted phosphorylation sites and the expression pattern of *kaiC* proteins in *M. macrosporus* and *M. xanthus*. **A** Multiple-sequence alignment between cyanobacterial (including *S. elongatus*) and myxobacterial KaiC protein homologs with arrows indicating two conserved phosphorylation sites of cyanobacterial KaiC. Multiple partitions are shown in the order as mentioned: both conserved sites in cyanobacterial homologs, both conserved sites in myxobacterial homologs, one conserved site in myxobacterial homologs, and lack of conserved site in myxobacterial homologs. **B** Expression of *kaiC* homologs in *M. macrosporus* and *M. xanthus* cultivated in light versus darkness. LILAB denotes genes originating from *M. macrosporus,* MXAN denotes genes originating from *M. xanthus*

In *M. xanthus,* all three *kaiC* genes are expressed during the developmental time course [50] and in the peripheral rods [42]. Our RNA-Seq analysis of liquid cultures of *M. xanthus* and *M. macrosporus*, containing vegetative cells revealed that all *kaiC* homologs are expressed independently of light-exposure (Fig. 7B). However, each homolog is differentially expressed when compared to others, with the highest values for LILAB_05235, followed by LILAB_13415, LILAB_10655, LILAB_22645, and LILAB_23600 in *M. macrosporus*. Nevertheless, interspecies comparison revealed differential gene expression between phylogenetically related *M. xanthus* and *M. macrosporus kaiC* genes (Fig. 7B). Two *M. macrosporus kaiC* orthologs, LILAB_05235 and LILAB_13415, showed higher expression when compared to *M. xanthus kaiC* orthologs (Fig. 7B). The third phylogenetically related ortholog LILAB_23600 had lower expression in *M. macrosporus* (Fig. 7B).

To identify potential protein interaction networks of *M. macrosporus* KaiCs, we implemented the STRING database [51, 52]. The STRING database revealed that all five KaiC homologs interact with the BphP in *M. macrosporus* (Fig. S6). Protein–protein interactions in STRING are derived from five main sources: Genomic Context Predictions, High-throughput Lab Experiments, Co-Expression Experiments, Automated Textmining, and Previous Knowledge in Databases. Nodes represent proteins, and color-coded lines represent potential interactions, highlighting a functional link between *M. macrosporus* KaiC homologs and BphP [53–59].

## 4 Discussion

Our findings highlight how light regulates different stages of the complex life cycle of myxobacteria and reveal the potential that BphPs are involved in the formation of fruiting bodies. Specifically, the fruiting-body development of *M. macrosporus* HW-1 influenced by light and trace amounts of nutrients, is likely to involve a complex molecular mechanism. Our results suggest that the molecular mechanism may involve interaction of BphP and circadian clock proteins, similar to plants, due to the unusual rhythmic behavior of fruiting-body formation of *M. macrosporus*. Explaining this cyclical behavior in non-photosynthetic myxobacteria could help us to understand how circadian rhythms influence other non-photosynthetic microorganisms. RNA-Seq analysis revealed that the genes in the BphP operon of *M. macrosporus* are constitutively expressed regardless of dark and/or light conditions. Detailed analysis of the downstream genes that may be regulated by the two-component signaling pathway of the BphP and the corresponding RR await further analysis. In the plant pathogen, *P. syringae*, the BphP has been demonstrated as the master regulator of swarming motility and several other complex behaviors. However, the BphP was not acting alone, and it worked in tandem with another, blue light sensing protein, LOV-HK [60]. Interestingly, in some myxobacteria, putative genes encoding another blue light photoreceptor, PYP (photoactive yellow protein), have been identified. Although present in *S. aurantiaca*, genes encoding PYPs are absent in *M. macrosporus* and *M. xanthus*.

The BphP of *M. macrosporus* may interact with circadian clock protein homologs. The multiple *kaiC* homologs, all expressed in various conditions and cell types of *M. xanthus* and *M. macrosporus,* are likely to serve an important function in myxobacteria. The cyanobacterial circadian clock, comprised of KaiABC proteins, is based on post-translational modification and can be recreated in vitro by mixing three purified Kai proteins with ATP [61]. In *S. elongatus,* KaiC is organized as a homohexameric ATPase with autokinase and autophosphatase activities. Changes in the phosphorylation state have been proposed to switch KaiC’s activity between autokinase and autophosphatase [62]. In addition, two homohexameric rings create the structure resembling a double doughnut with a central pore [63]. *S. elongatus* genome encodes a single copy of *kaiC*, in contrast to myxobacteria. It is possible that different homologs of myxobacterial *kaiC*s create heterohexamers, however we can only speculate without actual structure determination of these proteins. *S. elongatus kaiC* is rhythmically expressed with higher expression during the day, and decreased abundance at nighttime [64]. Our experiments did not detect significant differences in expression of myxobacterial *kaiC*s between light- and dark-cultivated cultures, which would suggest post-transcriptional regulation of the activity of KaiC by additional modulators, yet to be identified. Since the cyanobacterial circadian clock depends on post-translational modifications, lack of differential expression in *kaiC* gene does not dispute the possibility of a functional myxobacterial circadian clock.

The development of genetic tools is necessary to inactivate genes coding for BphPs and observe any changes in the fruiting-body morphology under various light conditions in many of these myxobacteral genera and species. Since *M. macrosporus* has one BphP gene, we could utilize the existing genetic tools of the closely related *M. xanthus* to inactivate this gene. BphPs have been implicated in the fruiting-body formation of related *S. aurantiaca* [6], although *S. aurantiaca* does not form concentric rings when cultivated in the same conditions as *M. macrosporus*. Recently, single particle cryo-EM studies of an *S. aurantiaca* BphP demonstrated how light regulates the differential HK activity [14]. The protein forms a heterodimer in the presence of ambient white light that is distinctly different from the protein homodimer captured upon illumination with red or far-red light. The authors propose that this heterodimer is as a signaling intermediate with one monomer in the Pr and another in the Pfr state. Furthermore, autophosphorylation assays using ATP (γ−^32^P) show HK activity of SaBphP in the presence of white light although the HK is less active in the presence of far-red light (740 nm) in comparison to red light (660 nm). Since the HK enzymatic activity of BphPs is regulated by light, constitutive expression of BphPs, including related *M. macrosporus* BphP between light and dark conditions would be expected. Concentric rings created by fruiting bodies are probably a result of peripheral rods motility. *M. xanthus* DK1622 is known for gliding motility (adventurous and social motility) [46] powered by Type IV pili (T4P). T4P are surface-exposed appendages playing a key role in myxobacterial motility and development. *M. xanthus* encodes 17 type IVa (T4aP) genes organized in a single cluster plus additional genes (distributed throughout the genome) for social motility and development [65]. Moreover, T4P-cluster architecture is the same in *M. macrosporus* and *M. xanthus* [65]. Energy sources, such as non-characterized nutrients from the bactoagar or casamino acids, are essential to generate ATP required for motility of *M. macrosporus*, and creation of additional fruiting bodies outside of the original inoculation. The observed differences in the fruiting-body arrangement between the regular concentric rings created on bactoagar and the random organization when casamino acids were added to the purified agar could be explained by the nutrient-dependent mechanism driving the phenomenon of ring formation. Interestingly, the circadian clock of *B. subtilis* is nutrient dependent [30]. Similarly, *M. macrosporus* may have a time-measuring mechanism that is not only light sensitive, but nutrient dependent as well.

RNA-Seq experiments were performed in different growth conditions involving liquid media with abundant nutrients thus direct comparison to fruiting-body formation on solid, starvation media is not possible. Nevertheless, these results suggest that the regulation of spatial photomorphogenesis is complex and depends on light, nutrient availability and/or growth substrate. Similar geometric phenotype was observed for other gram-negative bacteria and were modeled mathematically [66], involving limiting concentration of succinate and aspartate. This suggests that *M. macrosporus* may utilize limiting concentrations of amino acids in the bactoagar to fuel its motility in search for additional nutrients. Colonies of *E. coli* or *S. typhimurium* form geometrically complex patterns when exposed to, or feeding on, intermediates of citric acid cycle [67, 68]. In response to the citric acid cycle intermediate, the bacteria secrete aspartate, which is a potent chemo-attractant. Subsequently, the cells form high-density aggregates arranged in a regular pattern, by means of at least three very different pattern-forming processes. Although each pattern-forming process is unique, the essential elements such as, aspartate (chemo-attractant) and citric-cycle intermediate (pattern stimulant, such as succinate) remain the same. It is very possible that *M. macrosporus* uses aspartate as chemo-attractant. However, in this case we are examining rhythmicity of fruiting-body formation involving starvation-induced complex development of myxobacteria, distinctly different from nutrient-rich vegetative cells of *E. coli* and *S. typhimurium*.

## Supplementary Material

Supplementary Figures

## Data Availability

The data that support the findings of this study are available from the corresponding authors, EAS or MS, upon reasonable request.
