# Supplementary material for: Photomorphogenesis of Myxococcus macrosporus: new insights for light-regulation of cell development
Source: Photochem Photobiol Sci. Author manuscript; Available in PMC 2025 Feb 17. (PMC11832031; doi:10.1007/s43630-024-00635-1)
Supplement: Supplementary Figures [file NIHMS2049306-supplement-Supplementary_Figures.pdf]

Supplementary materials

Photomorphogenesis of *Myxococcus macrosporus*: New Insights for Light-Regulation of Cell Development

Kinga B. Graniczkowska[1], Dorina Bizhga[2], Moraima Noda[2], Viridiana Leon[2], Niharika Saraf[3], Denisse Feliz[2], Gaurav Sharma[3], Angela C. Nugent[2], Mitchell Singer[1] and Emina A. Stojković[2]

[1]Department of Microbiology and Molecular Genetics, College of Biological Sciences, University of California-Davis, CA, 95616, USA
[2]Department of Biology, Northeastern Illinois University, 5500 N. St. Louis Ave., Chicago, IL, 60625, USA
[3]Department of Biotechnology, Indian Institute of Technology Hyderabad, Telangana, India

corresponding authors: mhsinger@ucdavis.edu; e-stojkovic@neiu.edu

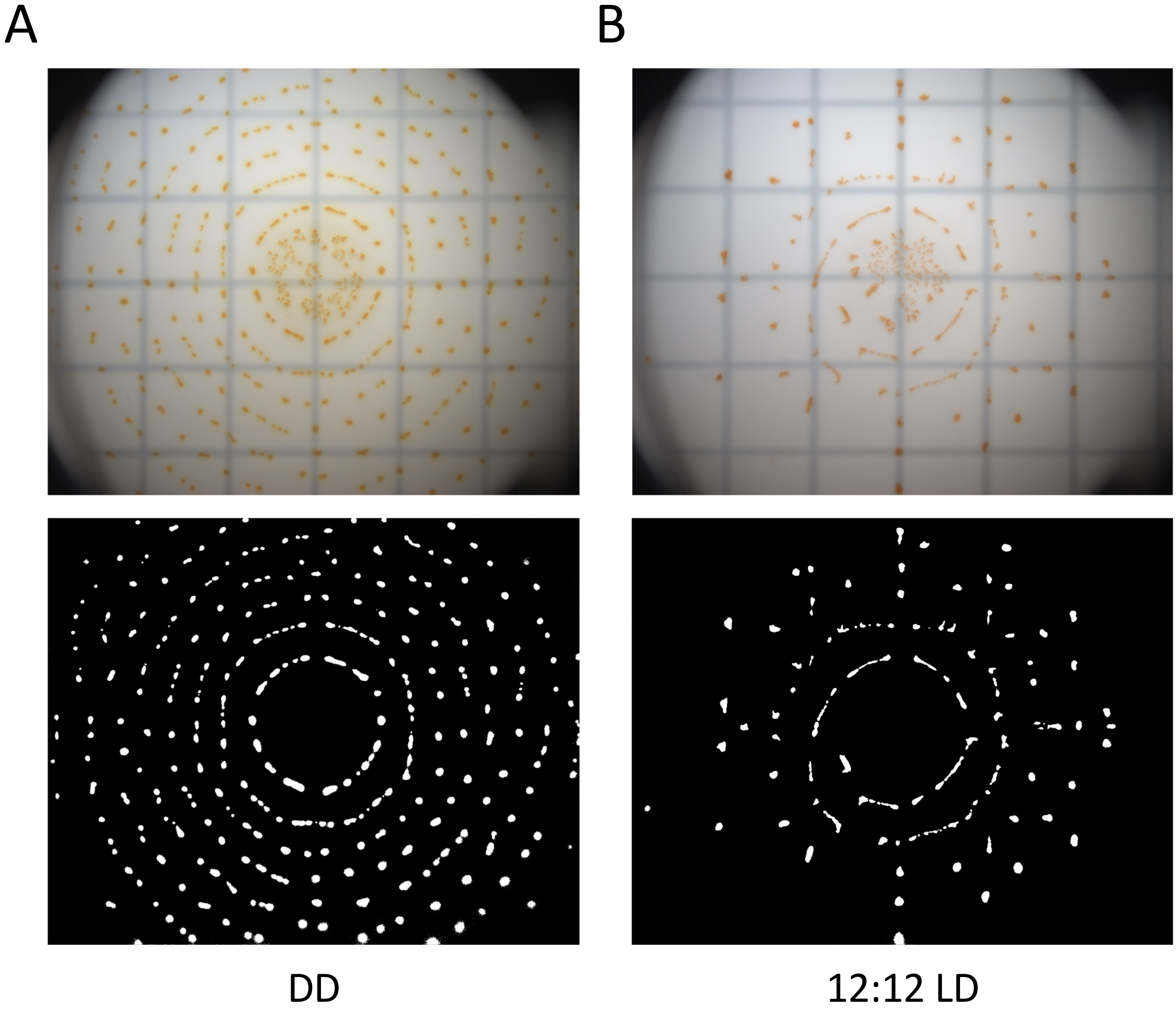

Supplementary Fig. S1 *M. macrosporus* fruiting bodies cultivated on filter paper placed on the Wasseragar (1.5%). A) constant darkness; B) 12:12 light-dark (LD) cycle. Images were taken on day 14 post inoculation. Bottom panels were converted into binary images using ImageJ. Grid from the filter paper has 3x3 mm dimensions.

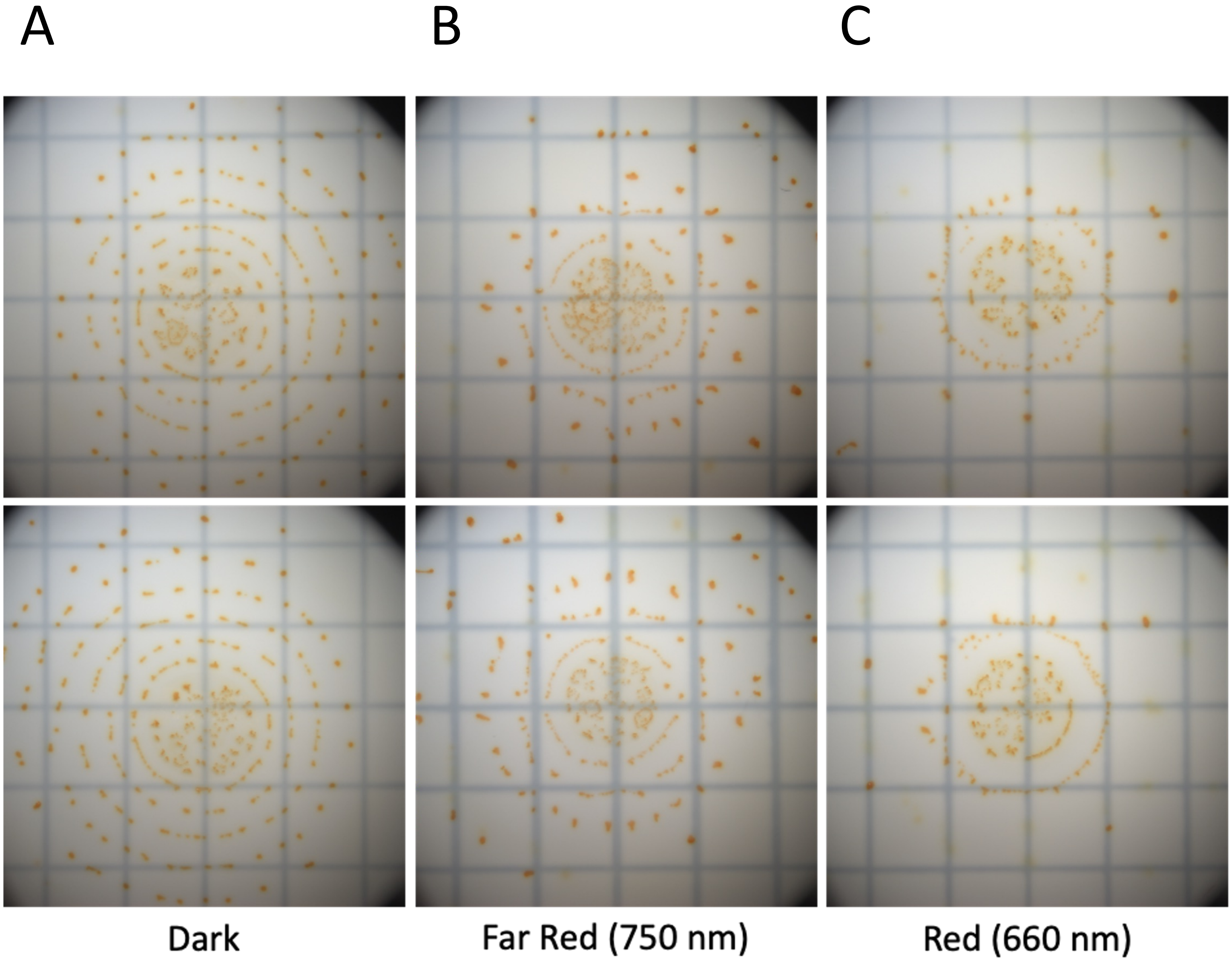

Supplementary Fig. S2 Differences in *M. macrosporus* fruiting bodies depending on the light conditions. Fruiting bodies were cultivated on filter paper placed on Wasseragar (1.5%), (A) constant darkness; (B) far red light (750 nm); and (C) red light (660 nm). Images were taken on day 14 post inoculation. Grid from the filter paper has 3x3 mm dimensions. Each condition is depicted in duplicates.

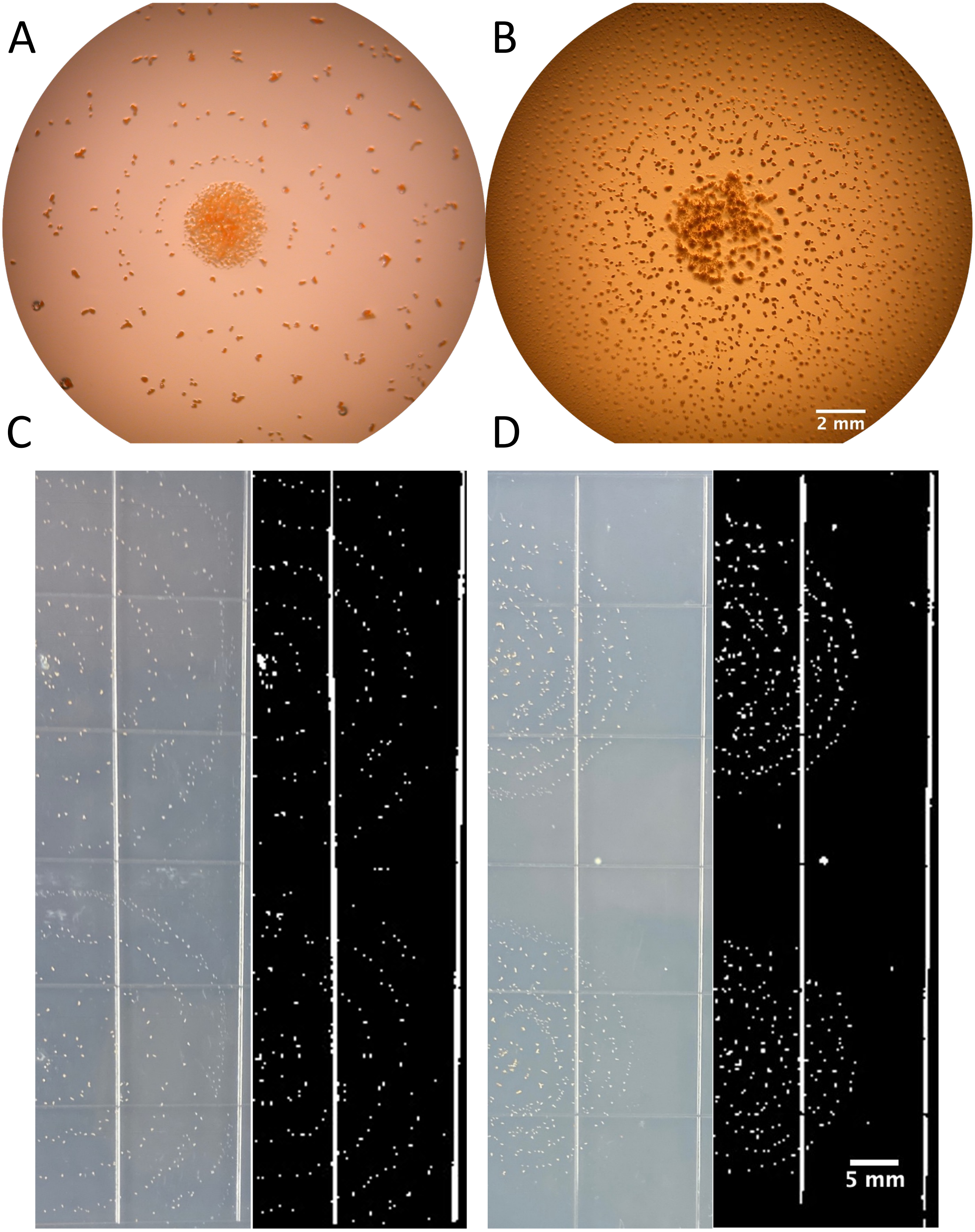

Supplementary Fig. S3 *M. macrosporus* fruiting bodies cultivated on Wasseragar prepared with A) 2% bactoagar, B) 5% bactoagar, C) 0.75% bactoagar, right panel was converted into binary image, and D) 1.5% bactoagar, right panel was converted into binary image. Differences in the agar color are the result of various agar concentrations. Images were taken on day 14 post inoculation.

Supplementary Fig. S4

Location of two phosphosites within *S. elongatus* KaiC protein

```
                                   S431 T432
                                     ↓  ↓
Consensus/70%                     lchs...slShlsDNllbLR blELctpl..
Synechococcus_elongatus_PCC7942   HSITD-SHISTITDTIILLQ YVEIRGEM--
Synechocystis KaiC3               NEQTE-VGVSSLMDTWLELQ TLRINGER--
Rhodopseudomonas_palustris        TE-TD-AGLSSLMDAWILLL NREAHGEF--
Synechocystis_KaiC2               HSITE-SHISTITDTILMLQ YVEIRGEM--
Synechocystis_KaiC1               ASITD-AHISTITDSIILLR YVEMYGEM--
Legionella_pneumophila            -ELTI-LGLSSLTETWIRLS NVESNGEF—

ArCb__BO221_RS35065               VRLPI-SGISSLVENIFFLR FVELHSKL--
ArCb__BO221_RS13125               -DPAA-PYLSTIPDAILVMD YARKPEGL--
Cyvi__Q664_RS12045                VVLPI-SGISSLVENIFFLR FVELHSKL--
Ag2261  AA314 RS00745             VKLPI-SGVSSLVENIFFLR FVELHAQL--
Ag2261__AA314_RS07270             -DAAP-PYLSTIPDAILLMD YARKEHGL--
Cyvi__Q664_RS24075                -DAAA-PYLSTIPDAILVMD YARKPEGL--
Ne__NAEX_RS11890                  VEATE-QNISSLIDTWILLR DVELGGER--
Ho__HOCH_RS15255                  LEATN-TAISSIVDTWLLLK TIELAGER--
Ho__HOCH_RS33420                  -DSQA-PYLSTLADAILMLG YDIRKDGL--

MxDK1622__MXAN_RS03430            LDVPMEVGPSAVAENLFFLR HVELEGRL--
MxDK1622__MXAN_RS32040            VTFPM-RGISMVAENILFLR QAELDSRL--
MxDZ2__MXDZ_RS0217380             LDVPMEVGPSAVAENLFFLR HVELEGRL--
MxDZ2  MXDZ RS0215800             VTFPM-RGISMVAENILFLR QAELDSRL--
Myma__MYMAC_RS14735               LDFSD-APVSILGENLLLLR YVELRGRI--
Myma__MYMAC_RS03655               LDVPMEVGPSAVAENLFFLR HVELEGRL--
Myma__MYMAC_RS31850               VAFPM-KGISMVAENVLFLR QAELDSEL--
MxDZF1__MXF1_RS0130255            LDVPMEVGPSAVAENLFFLR HVELEGRL--
MxDZF1  MXF1 RS0107090            VTFPM-RGISMVAENILFLR QAELDSRL--
Mh__A176_RS01315                  VRFPM-KGISMVAENVLYVR HAELDARL--
Mh__A176_RS30510                  LDVPMEVGPSAVAENLFFLR HVELEGRL--
Mh__A176_RS18510                  VDAPQ-PDATAYVENVILLR YVELRSQL--
Mx16526__BLV74_RS08075            LDVPMEVGPSAVAENLFFLR HVELEGRL--
Mx16526  BLV74 RS03525            VTFPM-RGISMVAENILFLR QAELDSRL--
Myvi__BLU09_RS28035               LDFSD-APVSILGENLLLLR YVELRGRI--
Myvi__BLU09_RS04590               LDVPMEVGPSAVAENLFFLR HVELEGRL--
Myvi__BLU09_RS08805               VTFPM-KGISMVAENILFLR QAELDSRL--
MfB__MFUL124B02_RS13600           VDAPQ-PDATAYVENVIMLR YVELRSQL--
MfB  MFUL124B02 RS03905           LDVPLESGPSAVAENLFFLR HVELEGRL--
Mebo__MEBOL_RS40705               LQFPL-QGLSMVAENTLYLR SVEMRAQL--
Mf__LILAB_RS13160                 VAFPM-KGISMVAENVLFLR QAELDSEL--
Mf__LILAB_RS22250                 LDFSD-APVSILGENLLLLR YVELRGRI--
Mf__LILAB_RS05140                 LDVPMEVGPSAVAENLFFLR HVELEGRL--
Sa__STAUR_RS03045                 IETPV-KGLSALVENHLFLR QVEWKGEL--
Sa__STAUR_RS21125                 L---E-GQISALSDAILSLQ VERPQNRS--
Sa__STAUR_RS02540                 VKLPM-QGISMVAENILFLR SVELHSEL--
Se__BMW77_RS17645                 VKFPM-RGISMVAENILFLR SVELNSEL--
Se__BMW77_RS17250                 IETPV-KGLSALVENHLFLR QVEWKGEL--
Se__BMW77_RS09025                 VDTPH-PEESAYVENIILLR YVELRSQL--
Se__BMW77_RS02425                 L---E-GQISALSDAILHLQ IERLGNRS--
Cfer__BON30_RS16195               LKFPL-QGLSLVAENILFLR SVELRSQL--
Cfer__BON30_RS33495               T-ISE-RGLSPLADNILMLR YLQDEGGL--
Cyb52655__CYFUS_RS50015           T-LSE-RGLSPLADNIFMLR YLQEGAGL--
Cyb52655__CYFUS_RS30790           LKFPL-QGLSLVAENILYLR SVELRSQL--
Cyb__D187_RS16130                 LDLAP-VGLSAGVDNIVFMR YVELRSQL--
Cyb__D187_RS05610                 T-ISE-RGLSPLADNIFMLR YLQDDGGL--
Cyb__D187_RS27140                 LKFPL-QGLSLVAENILYLR SVELRSQL--
Cc__COCOR_RS02965                 MDVPLGTGLSGVAENLLFMR HLELNGRL--
Cc__COCOR_RS19835                 VRFPL-KGISMVAENILFLR MVELHSGL--
Ag2261__AA314_RS22990             LGLAP-VGLSACVENIVFMR YVELRSQF--
Ag2261__AA314_RS13710             I-ISE-RGLSPLADNILMFR YVQEDSGL--
Hm__DB31_RS27050                  VETPV-KGLSALVENNIFLR QIELRGEL--
Hm__DB31_RS40585                  LTLPV-RGVSMLCENLFFLR SVEVRAEL--
Viti__DAT35_RS49955               VSFPM-KGISMVAENVLFMR AVELNSEL--
Ad1__A2CP1_RS15795                ML-AP-ADVTYLADTVVLLR YFEAGGGV--
AdC  ADEH RS16440                 -QLSG-HGVSFASDNVVQLR YVELGGRL--
AdC__ADEH_RS15235                 MV-AP-ADVTYLADTVVLLR YFEASGGV--
AsK__ANAEK_RS15240                ML-AP-ADVTYLADTVVLLR YFEAGGGV--
AsF__ANAE109_RS16580              SE-SP-FNISYIADTVLLFR YFEVAGEV—
```

```
Ho__HOCH_RS11995                FSATG-AGLSYMADTVVFLR YLELRGEL--
Ne__NAEX_RS33475                GQAPG-PDVSYLSDSIVVLG FYEIRGKI--
Ne__NAEX_RS07995                GR-SD-VDLSYLSDSLLLFR YFEARGRL--
Soce26  SOCE26 RS04900          INFSP-HSISFLAHNIIFLR YAEIDGQL--
Soce26__SOCE26_RS08750          AL-VR-LDISYLSDTVLLLS PFEFKGEM--
Soce26__SOCE26_RS28235          GEVPN-VDVSYLSDSILALR FFEAEGRI--

Mf__LILAB_RS10445               ADA-R-LGMETLVDNVLVLR LVALRSRH--
Mh  A176 RS19440                LDFSD-APVATLGENLLLLR YVELRGRI--
Cc__COCOR_RS29135               LDFND-APVASLGENLLLLR YVELHGQM--
Cc__COCOR_RS25900               VDMPQ-PEAASTVENVILVR YVELRSQL--

Cyvi  Q664 RS23675               LHSPI-KGAEAIFDNLIFLR FVELNGRL--
Cyvi__Q664_RS50095              LDFND-TPIAILGENLMLLR YVELRGRI--
Pp__PPSIR1_RS14825              --VGG-EAMVHTADAVFLIE EMGLTSKD(9
Cfer__BON30_RS36770             EEGPL-QGLEALIDNIVFLR YVELRSRL--
Cc__COCOR_RS37350               LHSPI-RGIEAICDNLVFLR FFELHGKL--
Soce0157  SCE1572 RS47355       IRMPN-PELGNVVETVVLLR YVELRSQI--
Cfer__BON30_RS39910             LDFND-SPIAMLGENLVLLR YVELRGRL--
Soce26__SOCE26_RS04890          LDFAG-SPLDVLAENVIFLR YVEFRGAL--
Ccmc__CMC5_RS39045              IQLPD-EVLAAVVENILVLR HVEVGSQL--
Es15201__DB30_02192             --VGG-EAMQHTADAVFLIE EMGLSSKE(9
Cyb  D187 RS22170                LVLPT-PELANVAESVVLLR YVELRSQI--
Soce26__SOCE26_RS47025          IKMPN-AELGNVVENVILLR YVELRSQI--
Cyb__D187_RS26250               EEGPL-QGLEALIDNIVFLR YVELRSRL--
ArCb__BO221_RS02265             LNLPT-PELANVVESVLLLR YVELRSQI--
Cap__CAP_RS08755                IKLPD-EDLAAVVENILVLR NVEVRSQL--
Hm  DB31 RS29830                 VETPL-AESAAYVENIILLR YVELRSQL--
Cfer__BON30_RS17430             LVLPT-PELANVAESVVLLR YVELRSQI--
Hm__DB31_RS20990                LDFSD-SPLANIAENLLFLR HVELRGRL--
Ag2261__AA314_RS43405           LNLPT-PELANVVETVLLLR YVELRSQI--
Sa__STAUR_RS22350               LDFND-TPIAMLGENLVLLR YVELRGRI--
Mebo  MEBOL RS20055              LHSPI-RGAEAIIDNLIFLR FVELNGRL--
Cyb__D187_RS24065               LDFND-SPIALLGENLVLLR YVELRGRI--
Mebo__MEBOL_RS14370             HEGPL-LGLEALIDNVVLLR YVEQRSQT--
Myma__MYMAC_RS34535             ADA-R-LGMEALVDNVLVLR LVALRSRH--
Myma__MYMAC_RS15695             VDSPQ-PDAAAYVENVILLR YVELRSQL--
Viti  DAT35 RS15410              LEAKL-EGLEAIVDNIVFLR YVELRSQL--
MxDZF1__MXF1_RS0123175          ADA-R-PDVEALVDNVLVLR MVALRSRN--
Sa__STAUR_RS03105               LHTPL-EGFEAIIDNLLFLR FVELRSQL--
Myvi__BLU09_RS30615             ADA-R-LDVEALVDNVLVLR MVALRSRN--
Ag2261__AA314_RS48465           LEAKL-EGVEGIVDNIVFLR YVEFRSQL--
Cyb52655__CYFUS_RS02295         EEGPF-QGLEALIDNIVFLR YVELRSRL--
Sa__STAUR_RS17185               VDSPH-PEEAAYVENIILLR YVELRSQL--
Sa__STAUR_RS21130               -------------------- ----------
Ccmc__CMC5_RS12705              --VGG-EALQHTADVVLLIE EMSLGSKE(9
Ag2261__AA314_RS13545           LDFSD-TPIAILGENLLLLR YVELRGRI--
Cyvi__Q664_RS37120              LNLPT-PELANVVESVLLLR YVELRSQI--
Mh__A176_RS35805                GDVLP-QGVEVLVDTILELR QVDLRSRR--
MxDZ2__MXDZ_RS0212030           ADA-R-PDVEALVDNVLVLR MVALRSRN--
Mf__LILAB_RS23230               VDSPQ-PDAAAYVENVILLR YVELRSQL--
ArCb__BO221_RS38100             LEAKV-EGVEGIVDNIVFLR YVEFRSQL--
ArCb__BO221_RS51885             LHSPI-KGAEAIFDNLIFLR FVELNGRL--
Cap__CAP_RS46190                --VGG-EALQHTADVVFLIE EMSLGSKE(9
Cyvi__Q664_RS35925              LEAKL-EGVEGIVDNIVFLR YVEFRSQL--
Mx16526__BLV74_RS00920          ADA-R-PDVEALVDNVLVLR MVALRSRN--
Cyb52655__CYFUS_RS44130         LALPT-PELANVAESVVLLR YVELRSQI--
Mebo__MEBOL_RS09975             LNLPT-PELANVVETVLLLR YVEMRSQI--
Mir__A7982_06542                --VGG-EALMHTADVVFLIE EMSLGSKE(9
ArCb__BO221_RS18590             LDFND-TPIAILGENLMLLR YVELRGRI--
Mebo__MEBOL_RS20415             LDFSD-TPIALLAENVLLLR YVELRGRI--
MxDK1622__MXAN_RS34615          ADA-R-PDVEALVDNVLVLR MVALRSRN--
Cyb52655__CYFUS_RS04150         LDFND-SPIAMLGENLVLLR YVELRGRI--
```

Supplementary Fig. S4 Multiple sequence alignment between *S. elongatus* KaiC and myxobacterial KaiC homologs with arrows indicating two phospho-sites of cyanobacterial KaiC.

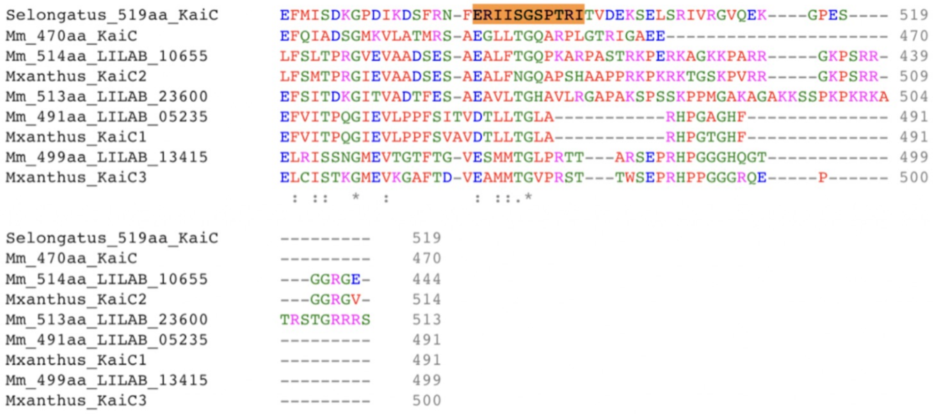

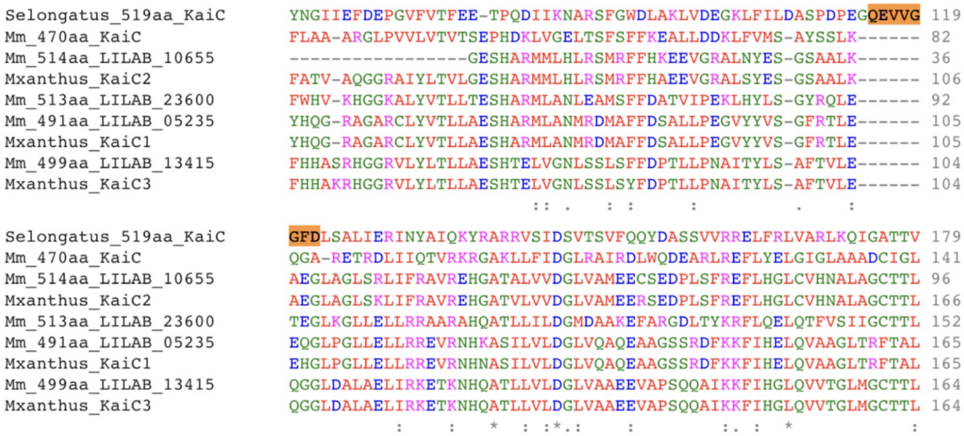

QEVVGGFD (B-loop, binding of KaiB)
ERIISGSPTRI (A-loop, binding of KaiA)

Supplementary Fig. S5 Multiple sequence alignment between *S. elongatus* KaiC and myxobacterial KaiC homologs with orange highlighting A-loop (binding of KaiA), and B-loop (binding of KaiB).

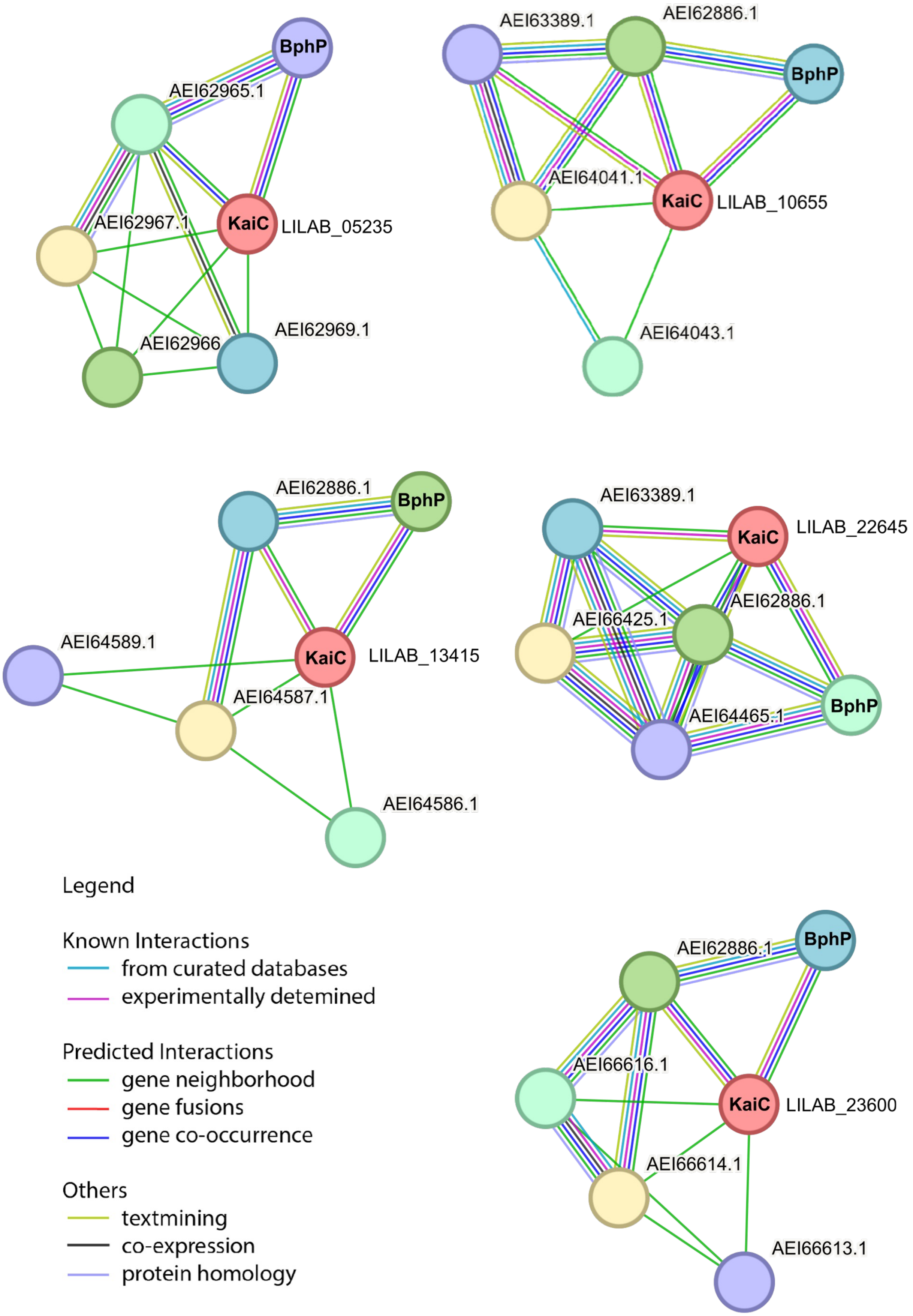

Supplementary Fig. S6 Predicted interactions between BphP and five KaiCs homologs. from *M. macrosporus* Interactions were predicted implementing STRING database (Szklarczyk et al. 2019; https://doi.org/10.1093/nar/gky1131).
